# Genomic Evidence for Rare Hybridization and Large Demographic Changes in the Evolutionary Histories of Four North American Dove Species

**DOI:** 10.3390/ani11092677

**Published:** 2021-09-13

**Authors:** Flor Hernández, Joshua I. Brown, Marissa Kaminski, Michael G. Harvey, Philip Lavretsky

**Affiliations:** Department of Biological Sciences, University of Texas at El Paso, El Paso, TX 79968, USA; fbhernandez2@miners.utep.edu (F.H.); jibrown@miners.utep.edu (J.I.B.); mkaminski@miners.utep.edu (M.K.); mgharvey@utep.edu (M.G.H.)

**Keywords:** secondary contact, Columbidae, demography, evolution, population genetics, speciation

## Abstract

**Simple Summary:**

Range expansions of invasive species continue to increase due to the direct or indirect influences of humans on global habitats. Understanding how these introductions and invasions increase the potential for interaction and hybridization between colonists and closely related native species is therefore increasingly important. We examine the evolutionary histories and signatures of hybridization among introduced feral Rock Pigeon and Eurasian Collared-Dove and native White-winged and Mourning doves in southwestern North America. Analyzing thousands of genomic markers, we find little evidence that hybridization has been extensive in their evolutionary histories or today. Despite this, evidence from multiple population genetics analyses supports the presence of six putative contemporary late-stage hybrids among the 182 sampled individuals. These putative hybrids all involve the most populous species, the Mourning Dove. We discuss the importance of using multiple marker types when attempting to infer complex evolutionary histories and propose important considerations when analyzing populations that were recently established or of domestic origins.

**Abstract:**

Introductions and invasions provide opportunities for interaction and hybridization between colonists and closely related native species. We investigate this phenomenon using the mitochondrial DNA COI and 81,416 base-pairs of overlapping nuclear variation to examine the evolutionary histories and signatures of hybridization among introduced feral Rock Pigeon and Eurasian Collared-Dove and native White-winged and Mourning doves in southwestern North America. First, we report all four species to be highly divergent across loci (overall pair-wise species *Φ_ST_* range = 0.17–0.70) and provide little evidence for gene flow at evolutionary timescales. Despite this, evidence from multiple population genetics analyses supports the presence of six putative contemporary late-stage hybrids among the 182 sampled individuals. These putative hybrids contain various ancestry combinations, but all involve the most populous species, the Mourning Dove. Next, we use a novel method to reconstruct demographic changes through time using partial genome sequence data. We identify recent, species-specific fluctuations in population size that are likely associated with changing environments since the Miocene and suggest that these fluctuations have influenced the genetic diversity of each dove species in ways that may impact their future persistence. Finally, we discuss the importance of using multiple marker types when attempting to infer complex evolutionary histories and propose important considerations when analyzing populations that were recently established or of domestic origins.

## 1. Introduction

Range expansions due to anthropogenic changes to the environment (e.g., species introductions or habitat destruction) have become a leading cause of secondary contact between many closely related native and non-native taxa [1,2,3]. Such contact events can result in competition with or the exclusion of native taxa [4]. If hybridization occurs, it may lead to adaptive introgression or increased diversity in the native species [5,6], but it can also facilitate the introgression of maladaptive traits into locally adapted populations [7,8]. If non-native species establish stable breeding populations and introgression into wild populations is pervasive, then local genetic variation can be swamped and eventually lost [7,9,10]. Therefore, it is important to understand the impacts of interaction and hybridization between native and anthropogenically introduced non-native taxa.

**Figure 1:**
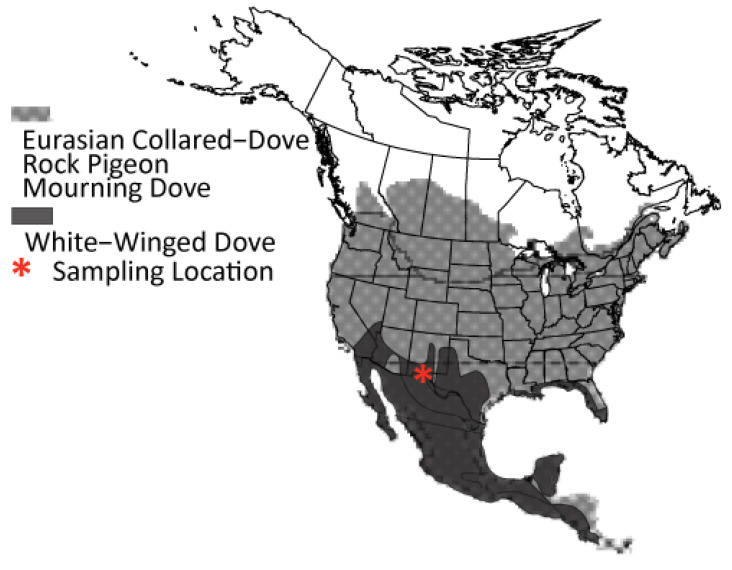
Geographic distribution of Rock Pigeon, Eurasian Collared-Dove, Mourning Dove and White-winged Dove. Star denotes sampling sites (sample specifics can be found in Supporting Information Table S1).

Many species in the pigeon and dove family (Aves; Columbidae) have experienced introductions or invasions. In North America, six of the ~54 occurring columbid (hereafter: “dove”) species have been introduced by humans [11]. Eurasian Collared-Dove (*Streptopelia decaocto*) and feral Rock Pigeon (*Columba livia domesticus*; a.k.a., “feral pigeon”), both native to Eurasia and northern Africa, are the most widespread of these and overlap widely with native populations of other doves such as Mourning (*Zenaida macroura*), and White-winged (*Zenaida asiatica*). Rock Pigeon was originally brought to North America by European settlers during the 1600’s [12], and has now established feral breeding populations throughout all of North America (Figure 1) [11,13,14]. Eurasian Collared-Dove was introduced more recently in the 1970’s [15,16], but has followed a similar trajectory and is now present across much of southern and western North America [17]. Although they are native to North America, White-winged Doves have also expanded through intentional releases by people [18,19], and due to increased urban land cover that has spurred a significant northward expansion out of Central Mexico over the last 60 years [13,20,21,22]. These range expansions have created broad overlap in the breeding and year-round ranges of Eurasian Collared, White-winged, and Mourning Doves and Rock Pigeons, most notably in the southwestern United States where all four overlap extensively [11,13,14]. This overlap may bring the four species into competition for resources that limits populations [23,24]. In addition, the potential for interspecific matings in the areas of overlap provides an opportunity for investigating the effects of contemporary gene flow from established non-native and feral populations on the genomes of native species.

Hybridization is frequent in many avian clades, with ~16% of species across all birds known to hybridize [25]. These high rates are likely a result of their strong dispersal ability [26], chromosomal stasis [27], and relatively slow development of reproductive isolation [28]. Although pre-zygotic barriers can be an early driver of avian reproductive isolation [29,30], post-zygotic barriers to reproduction (e.g., hybrid sterility) are extremely slow to develop in avian lineages [31,32,33]. Even intergeneric hybrids have been recorded in many avian families [34]. As a result, cases of allopatric speciation followed by secondary contact and hybridization are prevalent in birds [35,36,37,38]. Even though the large southwestern doves are not each other’s closest relatives [39,40], their recent extensive secondary contact following biogeographic histories of substantial allopatry may be producing opportunities for hybridization and introgression. There are isolated records of hybridization in Europe between Eurasian Collared-Dove and Rock Pigeon, in captivity between Eurasian Collared- and Mourning doves, and in captivity between Mourning Dove and Rock Pigeon [34]. There are also anecdotal records of putative Mourning and Eurasian Collared-Dove hybrids in the United States [41]. However, there has been no detailed investigation using genetic data into the presence or extent of hybridization in these species.

Our primary objective was to use detailed sampling and genome-wide data to evaluate the impact of population dynamics and gene flow on the evolutionary histories and current genetic diversity of the four large dove species in southwest North America. First, we estimated divergence across the genomes among all four species. Next, we tested for evidence of recent hybridization using a suite of methods. We then used coalescent analyses and model comparisons to evaluate the presence and rate of interspecific gene flow over evolutionary timescales. Finally, in a novel use of site-frequency spectrum based demographic analyses we reconstructed the effective population size of each species through time. We use results from these tests to discuss the history of genetic diversity and hybridization in these species and to project their future impacts on populations.

## 2. Materials and Methods

### 2.1. Sampling and DNA Extraction

From September–October of 2019, we opportunistically sampled 182 wing or breast tissues from hunter-shot Eurasian Collared-Dove (*n =* 29), feral Rock Pigeon (*n* = 33), Mourning Dove (*n* = 61), and White-winged Dove (*n* = 59) in southwestern New Mexico (Figure 1; Supporting Information Table S1). All sampled Rock Pigeon were considered to be feral as they displayed great variation in color and pattern, including brown and beige phenotypic coloration [42]. DNA was extracted using Qiagen’s DNeasy Blood & Tissue kit and following the manufacturer’s protocols (Qiagen, Valencia, CA, USA).

### 2.2. Mitochondrial DNA

A 500-base pair (bp) fragment of the mtDNA cytochrome oxidase subunit 1 (COI) gene was amplified and sequenced in Eurasian Collared-Dove, White-winged Dove, and Mourning Dove samples using newly designed primers DOVR (5′-GGTTKCGGTCYGTRAGTAG-3′) and DOVF (5′-RGGAGAYGACYMAATCTMYA-3′). Additional primers were designed for Rock Pigeon PigR (5′-AGGTTTCGGTCTGTGAGCAG-3′) and PigF (5′-CCTCCTCATCCGAGCAGAAC-3′). Mitchondrial COI sequences available in Genbank for each of the four dove species were aligned and locations of limited variability were targeted when designing primers. Fragments were amplified using an optimized touchdown PCR protocol on a 15 μL PCR solutions (see details in Supplementary Materials Document S1). Amplification was verified using gel electrophoresis with a 1% agarose gel, PCR products were cleaned using ExoSAP-IT^®^ (USB Corporation, Cleveland, OH, USA), and final products were sequenced on a 3130XL Genetic Analyzer at the University of Texas El Paso, Border Biomedical Research Center’s Genomic Analysis Core Facility. Raw Sanger sequences were aligned and edited using SEQUENCHER v4.8 (Gene Codes, Inc., Ann Arbor, MI, USA). All sequences have been submitted to GenBank (Accession Numbers OK086092–OK086272; Supporting Information Table S1).

### 2.3. ddRAD-Seq Library Preparation

ddRAD-seq libraries were created using Sbfl and EcoRI restriction enzymes, followed by ligating adapters containing sequences compatible for Illumina TruSeq reagents and barcodes for de-multiplexing. In general, ddRAD-seq protocols followed [43]; also see [36,44], but with size selection (mean size = 350 base-pairs; range 100–500 base-pairs) following a double-sided magnetic bead-based protocol developed here, and outlined in detail in Supporting Information Document S1. After ddRAD-seq library prep, all samples were pooled in equimolar amounts, and the multiplexed library was sequenced on an Illumina HiSeq X using single-end 150 bp chemistry at NovoGene (Novogene Co., Ltd., Sacramento, CA, USA). All raw Illumina reads are deposited in NCBI’s Sequence Read Archive (SRA; http://www.ncbi.nlm.nih.gov/sra accessed on 6 August 2021; BioPoject PRJNA761761; Bio Sample Accession Numbers SAMN21362192–SAMN21362373; Supporting Information Table S1).

### 2.4. Bioinformatics of ddRAD-Seq Data

Raw reads were de-multiplexed based on perfect barcode/index matches using the script *ddRADparser.py* [43]. We then used trimmomatic [45] to trim or discard poor quality sequences, with remaining quality reads then aligned to the Rock Pigeon reference genome (NCBI’s Sequence Read Archive data BioProject PRJNA347893: BioSample Accession Number SAMN02981415; [46]) ) using the Burrows Wheeler Aligner v. 07.15 (bwa, Auburn Hills, MI, USA [47]). Next, samples were sorted and indexed in Samtools v. 1.6 [48] and combined using the “mpileup” function with the following parameters “-c-A-Q 30-q 30” which set a base pair and overall sequence PHRED score of ≥30 to ensure that only high quality sequences are retained. All steps through “mpileup” were automated using a custom in-house Python script (Python scripts available at https://github.com/jonmohl/PopGen accessed on 6 August 2021; [44]). Next, VCF files for each marker, as well as concatenated autosomal and Z-sex chromosome markers, were converted to FASTA file formats using the program PGDspider v2.1.1.2 [49], with base-pair retention based on a minimum allele depth of 5× (i.e., 10× per genotype) and quality per base PHRED scores of ≥30. We then further filtered each FASTA file to remove base positions having <80% of alleles present (Python scripts available at https://github.com/jonmohl/PopGen accessed on 6 August 2021; [44]). We assessed allelic dropout by testing for Hardy-Weinberg Equilibrium across recovered loci and identifying loci with significant deviations based on a *p*-value of 1 × 10^−6^ [50] in VCFtools v. 0.115 ([51]; —hwe 0.000001), accounting for population structure using code from the dDocent bioinformatic pipeline [52].

Finally, sex was assigned to each sample based on the ratio of sequencing depth and heterozygosity at loci mapping to the Z chromosome relative to those mapping to autosomes. In short, given that females (ZW) and males (ZZ) are the hetero- and homogametic sex, respectively, Z-linked markers in females should appear homozygous and be recovered at about one half the sequencing depth of males. Importantly, we had access to full specimens of the Rock Pigeons and were able to additionally sex these by dissection, which served as a positive control for genetic sex assignments.

### 2.5. Phylogenetic Analyses

An mtDNA species tree was reconstructed in *Beast v2.6.0 [53] using a multispecies Calibrated Yule tree method (Species Tree: Yule Process), with a Mallard used to root the tree (GenBank Accession Num. MK262361.1 [54]). We determined the optimum base-pair substitution model in MEGA v10 [55], and tested between strict and variable molecular clocks by comparing Bayes Factors estimated from respective reconstructed species trees. The tree was time-calibrated based on the split time between Mallards and Rock Pigeons (93.2–104.6 mya [56]). Beast analyses were based on a total of 500 million MCMC generations with sampling every 5000 generations to ensure that effective sample size (ESS) values across estimated parameters were >50 (see details in Supplementary Materials Document S1). We note that multiple independent runs were done, with all of them converging to similar estimates and phylogenetic relationships, as well sufficient mixing among MCMC traces for each estimated perimeter. Consequently, the final species tree was based on the *Beast longest run.

Next, a nuclear species tree was reconstructed in the program TreeMix version 1.12 [57], and rooted with data from shared ddRAD-seq loci from a wild Mallard sequenced using the same ddRAD-seq protocol. Note that any putative contemporary hybrids identified in the structure analyses above were excluded from this and all subsequent tests of evolutionary history using nuclear DNA in order to reduce the impacts of very recent events on inference. TreeMix was also used to test for historical gene flow. Specifically, we simultaneously estimated a maximum likelihood (ML) species tree and the direction and weight (*w*) of gene flow among taxa based on allele frequencies. Analyses were run across each bi-allelic SNP (-k 1), with global rearrangement occurring during tree building (-global), and with nodal support based on 1000 bootstraps. The optimum number of migration edges was determined by sequentially adding migration events up to ten (−m 0–10), and then evaluating the proportion of the variance explained by each migration model. In order to limit overconfidence in the tree model, migration edges were added until >98% of the variance in the tree model was explained. Finally, likelihood ratios and standard errors (-se) were calculated to assess significance between tree models and migration edges, respectively (see details in Supplementary Materials Document S1).

### 2.6. Population Structure & Diversity Statistics

For mtDNA, we visualized population structure by creating a haplotype network in the program NETWORK v10.1 [58].

For nuclear DNA, population structure was based on bi-allelic ddRAD-seq autosomal SNPs. First, we performed Principle component analysis (PCA) as implemented in the package adegenet in R (i.e., “dudi.pca” [59]; also see [60]. Next, we calculated maximum-likelihood-based individual assignment probabilities using the program ADMIXTURE v1.3.0 [61,62]. We evaluated *K* population values of one through ten, with 100 runs at each *K*. The optimum *K* was based on the lowest average of CV-errors across 100 analyses per evaluated *K* value. Results were combined across runs using CLUMPP V. 1.1 [63]. Finally, we evaluated relationships and admixture among samples based on co-ancestry assignments in the program fineRADstructure [64]. All detailed population structure methods can be found in Supplementary Materials Document S1.

Values of nucleotide diversity (π) and pair-wise species *Φ_ST_* values were estimated across ddRAD-seq autosomal and Z-chromosome linked loci as well as mtDNA in VCFtools version 0.1.11 [50] and DnaSP 6 [65], respectively. Nuclear loci were plotted by genomic location to look at patterns of genomic divergence.

### 2.7. Effective Population Size, Divergence Time, and Migration Rates

For mtDNA, Isolation-with-Migration models as implemented in IM [66,67] were used to estimate effective population size, divergence time, and migration rates for pairwise comparisons of all four species (see specifics in Supporting Information Document S1). In short, IM simultaneously calculates posterior probability densities of population sizes, divergence time, and migration rates from non-recombinant sequence fragments using Bayesian MCMC algorithms [68].

For nuclear DNA, we used the program ∂a∂i [69,70], which implements a diffusion-based approach to test empirical data against specified evolutionary models (e.g., Isolation-with-Migration; also see detailed methods in Supporting Information Document S1). Briefly, ∂a∂i determines the best fit evolutionary model using a site-frequency spectrum derived across all base-pair positions. We tested three evolutionary models including Isolation-with-Migration, Split-Migration (i.e., recurring secondary contact), and Neutral-No-Divergence (all developed scripts can be found here: https://github.com/jibrown17/Dove_dadi.pairwise.comparisons accessed on 6 August 2021). We used the best fit model as determined by ∂a∂i to calculate the optimal parameter values as well as uncertainty metrics (i.e., standard deviation [69,71]).

Finally, to convert parameter estimates from IM and ∂a∂i into biologically informative values, we estimated generation time (*G*) and mutation rates per locus (*μ*; also see detailed methods in Supporting Information Document S1). We used an average mitochondrial mutation rate for birds of 1.035 × 10^−8^ substitutions/site/year [72]. For nuclear DNA, the mean avian nuclear mutation rate (1.2 × 10^−9^ substitutions/site/year [73]) is unlikely to be representative of our four species (i.e., Columbiformes tend to show slower mutation rates [74]) or the subset of the genome present in our ddRAD-seq markers. Instead, we calibrated ∂a∂i parameters to the average time determined in our *BEAST analysis of mtDNA for the split between the Eurasian (i.e., Eurasian Collard-Doves and Rock Pigeons) and North American (White-winged and Mourning Doves) clades. This provided a calibrated nuclear mutation rate that was then used to convert remaining ∂a∂i parameter values [75] (see details in Supplementary Materials Document S1). Mutation rates were then scaled to the generation time for each species and multiplied by the total number of base pairs for mtDNA (419 bp) and nuclear DNA (79,862 bp) to obtain respective mutation rates scaled to substitutions/site/generation (s/s/g).

### 2.8. Historical Population Demography through Time

Long-term demographic histories of each species were determined using a novel ∂a∂i model that estimates effective population size through time using partial genome sequence information (see details in Supplementary Materials Document S1). In short, using all recovered ddRAD-seq autosomal loci, we created a one-dimensional (i.e., single species) site-frequency spectrum (SFS) for each species where Nexus formatted SNP datasets are transformed into species-specific SFS using custom python scripts (all developed scripts can be found here: https://github.com/jibrown17/Dove_dadi.demographics accessed on 6 August 2021). The SFS was then folded and masked at sites with variants present in only one or all samples, and each dataset run through our custom model (all developed scripts can be found here: https://github.com/jibrown17/Dove_dadi.demographics accessed on 6 August 2021) where effective population size is estimated through a series of time intervals. Our stepwise time interval function uses 100 iterations of the single population integration function (‘*Integration.one_pop*’ in ∂a∂i) to model a continuous transformation of effective population size through time. The ancestral effective population size of *v_Anc_*, exists for some time-period, *T_Anc_*, before estimating the effective population size, *v_n_*, for some time interval, *T_n_*, at each subsequent integration step. Effective population size is then estimated for time intervals in the past, starting with *T*_0_, until the present day, *T*_99_, and the ancestral population will have occurred at time, *T*_99_ + *T*_98_ + *T*_97_ … + *T*_1_ + *T*_0_ + *T_Anc_*. This stepwise function is then used to model an SFS that is subsequently fit to the empirical data for each species through parameter optimization, which we then compare across 50 runs per species. Final optimal parameters are scaled to the empirical data using *θ* (*θ = 4N_ANC_* × *μ*; *N_ANC_* = Ancestral effective population size), and based on the obtained calibrated mutation rate (see above; see details in Supplementary Materials Document S1). Goodness of fit for each species’ model SFS was based on the log-likelihood of the model given the empirical data. Finally, we calculated the 95% confidence intervals (CI) using the parameter uncertainty metrics included in ∂a∂i (see details in Supplementary Materials Document S1). The effective population size and time parameters were converted into biologically informative numbers as previously described, and based on generation time (*G*), age of sexual maturity (*α*), survival, and the substitution rate calibrated with previously determined divergence estimates (see details in Supplementary Materials Document S1).

## 3. Results

A total of 419 base-pairs (bp) of overlapping mtDNA COI sequence was obtained across samples, with the exception of one Mourning Dove (Supporting Information Table S1). For ddRAD sequencing, a total of 377 million raw HiSeq Illumina reads were recovered across all samples. After quality filtering, a total of 81,416 base-pairs (bp) of overlapping sequence was recovered across 27 autosomal chromosomes (79,862 bp) and the Z-sex chromosome (1554 bp; Supporting Information Figure S1). Across sites, we recovered an average per sample median sequencing depth of 119 (per sample average depth range = 58 –148). We identified 24 of 81,416 (~0.03%) base-pair positions across 8 of 28 chromosomes that deviated from Hardy-Weinberg Equilibrium. Given the small number of SNPs involved, we conclude that our data filtering strategies were sufficient to limit allele dropout and did not exclude these SNPs from our final dataset. Finally, we were able to identify the sex of all individuals using the ratio of sequencing depth and heterozygosity between autosomal and Z-sex chromosome linked markers (Supporting Information Figure S2).

Nucleotide diversity differed among species and between marker types (Table 1). Whereas Mourning Dove showed a two- and three-fold higher nucleotide diversity at ddRAD-seq Z-sex and autosomal chromosomes, respectively, Eurasian Collared-Doves had three to four-fold higher calculated nucleotide diversity at mtDNA as compared to the remaining dove species.

### 3.1. Phylogenetics

**Figure 2:**
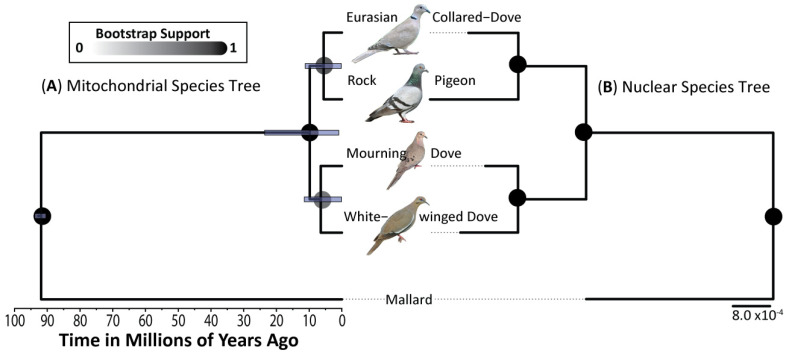
Species tree reconstruction of sampled Rock Pigeon, Eurasian Collared-Dove, Mourning Dove, and White-winged Dove, as well as a mallard that served as an outgroup (Supporting Information Table S1), and based on (**A**) a *BEAST analysis of 419 base-pairs of the cytochrome oxidase subunit 1 (COI) gene or (**B**) a TreeMix analysis of 11,053 independent ddRAD-seq autosomal bi-allelic SNPs and under an optimum model of no migration.

For mtDNA, a Hasegawa-Kishino-Yano (HKY) substitution model with a relaxed exponential clock (Bayes Factor = 3.30) was found to be the optimal model for the *BEAST species tree. The nuclear TreeMix species tree was reconstructed from allele frequencies calculated from 11,175 (of 11,856) independent bi-allelic ddRAD-seq autosomal SNPs that met our filtering criteria. A TreeMix species tree without gene flow was the optimum model and was found to explain >99% of the variance. Trees including migration edges (i.e., gene flow) were not statistically better (*X*^2^ critical value < 1), nor were any of the identified migration edges supported (*p*-value > 0.1). As expected, both trees recovered well-supported sister relationships between the two Eurasian (Rock Pigeon and Eurasian Collared-Dove) and two North American (Mourning and White-winged doves) species (Figure 2). Using a known divergence time between Anseriformes and Columbiformes of 93 million years before present [56], and an overall avian mtDNA mutation rate of 1.035 × 10^−8^ [76], we estimated a divergence time between Eurasian and North American clades at 9.8 Mya (95% Highest Posterior Density [HPD] = 0.95–23.59). Divergence time for species within the North American and Eurasian clades was 6.4 Mya (95% HPD = 0.15–11.49) and 5.6 Mya (95% HPD = 0.18–11.23), respectively (Figure 2A).

### 3.2. Population Structure & Recent Hybridization

**Figure 3:**
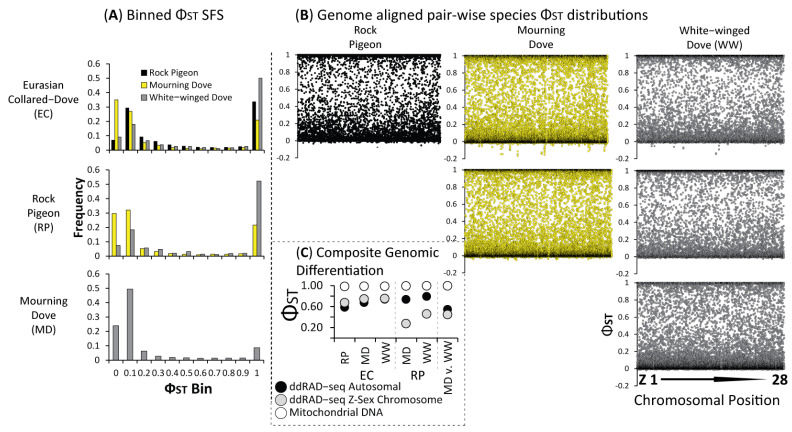
The (**A**) site-frequency spectrum (SFS) and (**B**) per chromosome location pair-wise relative differentiation (*Φ_ST_*) estimates between Rock Pigeon, Eurasian Collared-Dove, Mourning Dove and White-winged Dove estimated across the Z-sex and 27 autosomal chromosomes (also see Supporting Information Figure S1). We also provide (**C**) the composite pair-wise relative *Φ_ST_* estimates between the four dove species and across the mitochondrial cytochrome oxidase subunit 1 (COI) gene, as well as concatenated datasets of each ddRAD-seq autosomal and Z-sex chromosome linked loci.

**Figure 4:**
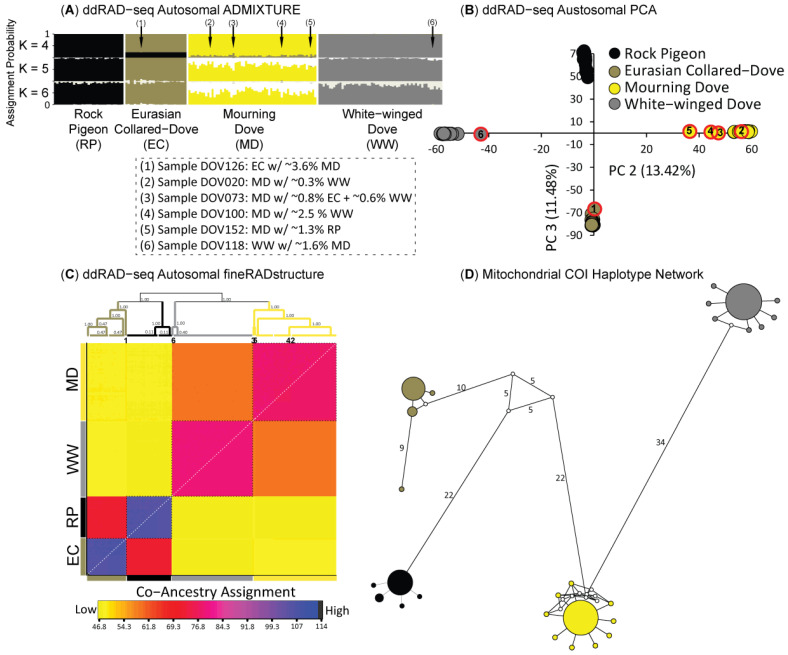
Individual assignments and clustering of sampled Rock Pigeon (RD), Eurasian Collared-Dove (EC), Mourning Dove (MD), and White-winged Dove (WW) that were based on a 11,175 bi-allelic ddRAD-seq autosomal SNP dataset, and visualized as (**A**) ADMIXTURE likelihood assignment probabilities based on *K* models of 4–6, (**B**) the first two components of the Principle component analysis (PCA), and (**C**) a matrix of individual (above the diagonal) and average (below the diagonal) co-ancestry coefficients along with the resulting dendrogram from the fineRADstructure analysis. Note that coancestry ranges from low (yellow) to high (blue) as indicated by the color scale. Inset identifies the six samples found to be admixed and provides their genetic constitutions as determined from ADMIXTURE analyses. The six hybrids are numerically denoted and their positions identified across analyses. The numbers correspond to the sample locations across nuclear based analyses (**A**–**C**). Finally, we visualize (**D**) mitochondrial DNA relationships through a median-joining network where size of circles corresponds to total number of individuals with that haplotype, and branch lengths indicate the number of mutations separating haplotypes; all branches with >1 mutation are noted.

The four dove species were highly divergent with elevated composite estimates of relative differentiation (*Φ_ST_*) for ddRAD-seq (Avg. Autosomal *Φ_ST_* = 0.68; Avg. ddRAD-seq Z-Sex Chromosome *Φ_ST_* = 0.56) and mtDNA (Avg. *Φ_ST_* = 0.99) markers (Figure 3C). Moreover, locus-by-locus estimates of *Φ_ST_* for ddRAD-seq loci showed large portions of the nuclear genome were fixed in pair-wise species comparisons (Figure 3A,B). To limit any issues due to heterogamy of the distinct evolutionary history of the Z-sex chromosome, further analyses of population structure and evolutionary history were based on either mtDNA or autosomal ddRAD-seq markers only. As expected given the nearly fixed estimates of *Φ_ST_* for mtDNA markers (*Φ_ST_* > 0.97; Figure 3C), we recovered reciprocal monophyly in our haplotype analysis, where no haplotypes were shared among the four dove species (Figure 4D). Among the four species, Mourning Dove (*N_haplotypes_* = 10) were most diverse for mtDNA COI haplotypes, followed by White-winged Dove (*N_haplotypes_* = 8), Rock Pigeon (*N_haplotypes_* = 5), and Eurasian Collared-Dove (*N_haplotypes_* = 4); all four species possessed minor haplotypes largely distinguished by a single mutation from their respective major haplotype (Figure 4D).

Further population genetic structure analyses included the same 11,175 independent bi-allelic ddRAD-seq autosomal SNPs as used in species tree analysis (Figure 2B). ADMIXTURE (Figure 4A), PCA (Figure 4B), and fineRADstructure (Figure 4C) analyses all identified four distinct clusters that correspond with species designations. Although ADMIXTURE analyses assigned all samples to their respective species with >95% assignment probability (Figure 4A), some individuals had low levels of mixed assignment probability. Further scrutiny of six of these individuals in fineRADstructure results revealed that they had elevated co-ancestry assignments to more than one species across the genome, which resulted in placement on isolated branches in the fineRADstructure dendrogram (Figure 4C). The individuals with mixed ancestry involved the following ancestry combinations: (1) Mourning Doves (*n* = 4) with some ancestry from White-winged Dove (*n* = 2), Rock Pigeon (*n* = 1), or a mix of White-winged Dove and Eurasian Collared-Dove (*n* = 1); (2) a single Eurasian Collared-Dove with some ancestry from Mourning Dove; and (3) one White-winged Dove with some ancestry from Mourning Dove (also see inset in Figure 4). In addition, these six individuals clustered away from their respective primary species in the PCA and closer to the cluster representing the putative “minor” source of ancestry (Figure 4B).

### 3.3. Optimum Evolutionary Models and Estimated Parameters

Though ESS values were ≥50 across all comparisons in mtDNA IM analyses, likelihood distributions for some parameters (ancestral effective population size and time since divergence) did not converge in any pair-wise species comparisons (Supporting Information Figures S3–S6). For nuDNA, ∂a∂i returned an optimum evolutionary model of Split-with-Migration for all but two comparisons. A Split-without-Migration was favored in the Rock Pigeon and Mourning Dove comparison, and an Isolation-with-Migration model in the White-winged Dove and Mourning Dove comparison (Supporting Information Table S2).

**Figure 5:**
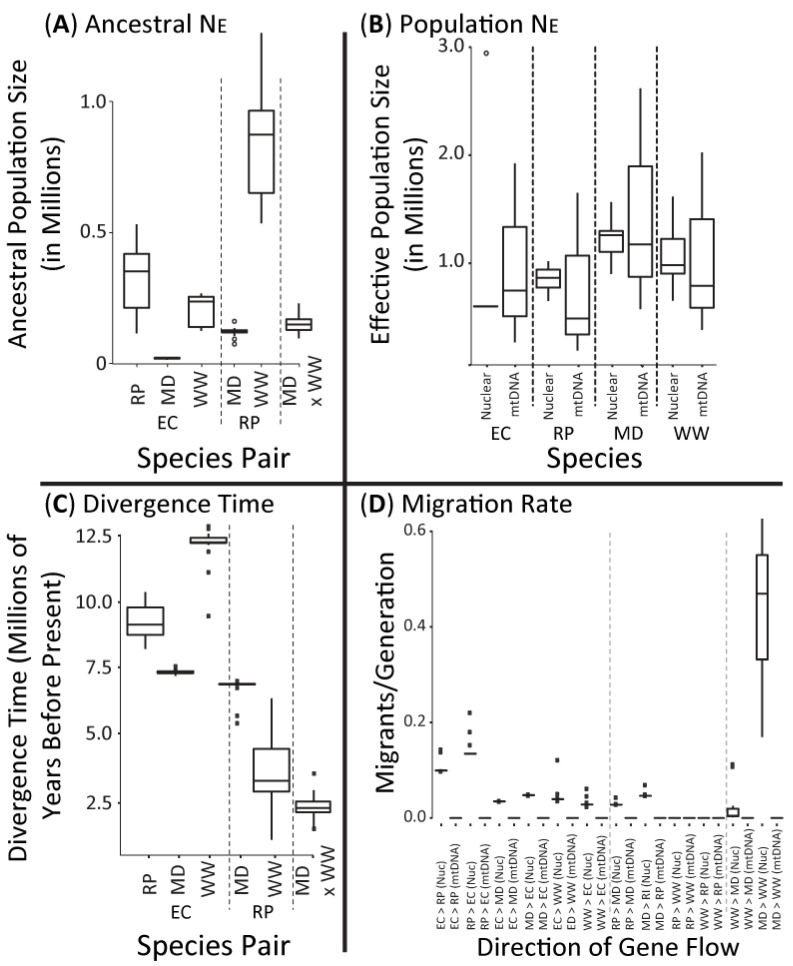
Box plots are of values across pair-wise species IM and/or ∂a∂i analyses of the mitochondrial (mtDNA) cytochrome oxidase subunit 1 gene or ddRAD-seq autosomal loci, respectively, and estimates of (**A**) ancestral effective population size, (**B**) effective population sizes of contemporary Eurasian Collared-Dove (EC), Rock Pigeon (RP), Mourning Dove (MD), and White-winged Dove (WW), (**C**) divergence time, and (**D**) migration rates (directionality is denoted as “from > to”). Note that IM analyses of mtDNA did not converge for ancestral effective population size and divergence times (Supporting Information Figures S3–S6); and thus, marker comparisons were only achieved for per-species effective population size and migration rates. For comparison, diamonds within contemporary size estimates (top right) denote the most recent effective population size as obtained from the per species time-series demographic analyses (i.e., Figure 6).

**Figure 6:**
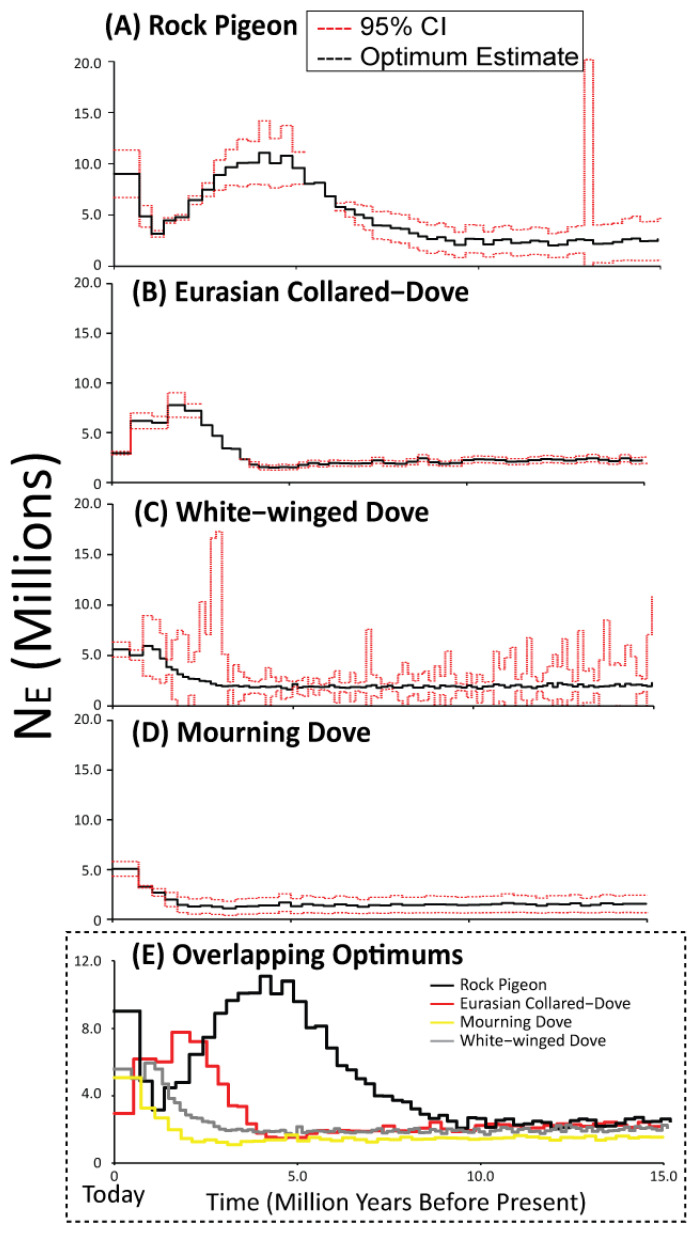
Per species time-series demographic analyses and associated 95% confidence intervals estimated using our developed single species ∂a∂i model, and based on bi-allelic ddRAD-seq autosomal SNPs for (**A**) Rock Pigeon, (**B**) Eurasian Collared-Dove, (**C**) Mourning Dove, and (**D**) White-winged Dove (**E**). We also provide overlapping optimum estimates across the four dove species for comparison.

Nuclear ∂a∂i parameters, when calibrated using divergence times estimated from the *Beast mtDNA species tree, included an average autosomal mutation rate of 1.95 × 10^−10^ mutations/site/year. We recovered similar ancestral population sizes across pairwise species analyses (Figure 5A) that were substantially lower than each species’ contemporary effective population size (Figure 5B), supporting the idea that all four species have undergone recent population expansion (also see Figure 6). All contemporary effective population sizes estimated from mtDNA and nuDNA had broadly overlapping confidence intervals, with Mourning Doves generally possessing the largest effective population sizes, followed by White-winged Dove, and near equal estimates for Rock Pigeon and Eurasian Collared-Dove (Figure 5B; Table 1). Consistent with reciprocal monophyly recovered in the mtDNA haplotype network (Figure 4D), posterior distributions for all pair-wise species IM analyses overlapped zero gene flow (Figure 5D). Conversely, nuclear-based ∂a∂i results indicated that an evolutionary model that incorporates gene flow was the best fit for five of the six species comparisons (Supplementary Materials Table S2); although all estimates of gene flow were < 1 migrant per generation (Migrants/generation = 0.00–0.22; Figure 5D). Finally, based on the applied mutation rate, we recovered a stepwise pattern of divergence times for nuclear DNA between the four dove species, with Mourning and White-winged Dove diverging most recently (Figure 5C).

### 3.4. Historical Population Sizes

Time-series estimates of effective population size using the novel ∂a∂i demographic models retained near identical estimates of effective population size (*N_e_* = 1.37–2.65 million individuals) prior to ~10 Mya (Figure 6E). Rock Pigeon was the first species to deviate approximately 9 Mya and steadily increased in size for five million years before peaking at 11.1 million individuals, it then declined until ~1.4 Mya before rapidly increasing to reach a contemporary effective population size of nine million (95% CI 6.71–11.35 million; Figure 6A). *Eurasian Collared-Dove* showed a similar trend, rapidly increasing from the ancestral state about four million years ago before showing declines over the last two million years to a contemporary effective population size of three million individuals (95% CI 2.77–3.12 million; Figure 6B). Finally, both Mourning and White-winged Dove deviated from the ancestral state ~3.5 Mya and have continued increasing, with contemporary effective population sizes of ~5 million (Mourning Dove 95% CI = 4.34–5.81 million; White-winged Dove 95% CI = 4.84–6.33 million; Figure 6C,D).

## 4. Discussion

### 4.1. Largely Allopatric Evolutionary Histories Lead to Highly Divergent Genomes among Four Dove Species

We identified population genetic structure and genome-wide divergence among the study species, with haplotypes from the sequenced COI portion of the mitogenome (Figure 4) and 10–60% (Figure 3) of the sampled nuclear genome fixed between species. Moreover, migration estimates over evolutionary time indicate little genomic exchange. All pair-wise species migration rates were <1 migrant per generation regardless of marker-type (Figure 5D), and we recovered an optimal TreeMix species tree that excluded gene flow (Figure 2B). It is important to note that the best-fit evolutionary models returned by ∂a∂i based on nuclear DNA for most pair-wise comparisons was Split-with-Migration, suggesting a history of isolation with potential bouts of limited gene flow during secondary contact event(s). However, the rates of gene flow estimated in ∂a∂i were very low. Together, these results are indicative of a prolonged and separate evolutionary history. This is consistent with the taxonomic treatment of these taxa in separate species and genera, with the biogeographic history of these species, and with prior phylogenetic work indicating deep divergence [39,77].

The divergence times from our analyses place the split between the Eurasian and North American taxa to the late and middle Miocene (Figure 2C and Figure 5). This is concordant with earlier work based on full mitogenomes [78]. We also note that the estimated divergence time of ~6.4 Mya between Mourning and White-winged Doves based on the mtDNA COI gene overlap those estimated previously from a few nuclear and mtDNA genes [39]. However, other divergence times estimated from our nuclear and mtDNA trees differ from one another and from previous work [78,79,80]. For example, the divergence time based on the sequenced mtDNA COI section that dates the Rock Pigeon and Eurasian Collared-Dove split to the late Miocene (i.e., ~5.6 Mya) is ~10 million years more recent than previous estimates [78,79,80]. Moreover, whereas we estimate a divergence time of ~6.4 Mya between Mourning and White-winged Doves based on the mtDNA, nuclear estimates are about half that regardless of analytical method (Figure 5B and Figure 6). Differences in time estimates may be the result of discordance among different marker types (i.e., nuclear versus mtDNA), a widespread phenomenon in birds [81,82,83,84,85,86]. Discrepancies may also stem from time calibration, which may fail to capture rate variation particular to different marker types. More accurate divergence time estimates will require further understanding of how mutation rates vary across the genomes and among lineages in Columbidae.

### 4.2. Limited Anthropogenic Gene Flow Today among Four Dove Species

Despite strong genomic divergence between the four species, we identified six individuals that are inferred to have mixed ancestry. All six, however, have low levels of ancestry (<5% assignment probability) from the “minor” species in ADMIXTURE and show only weak separation from species clusters observed in PCA results. These patterns could be indicative of individuals representing late-stage back-crossed hybrids or, alternatively, outlier individuals that have inherited elevated numbers of ancestral alleles. However, these individuals also clearly show co-ancestry with multiple species in the fineRADstructure co-ancestry matrices. fineRADstructure gives more weight to rare alleles and is more sensitive to low levels of admixture that are expected in backcrosses [64]. Taken together, these results support the identification of these individuals as putative late-stage backcrossed hybrids. While intergeneric hybrids are thought to be rare, they are widespread in many avian groups (e.g., [87,88,89]). Viable intergeneric hybrids involving Eurasian Collared-Doves, Rock Pigeons, and Mourning Doves have been found previously [34], and there are anecdotal records in public repositories of hybrids in North America, at least between Eurasian Collared- and Mourning doves [41]. Therefore, our identification of putative hybrids in population genomic data from these species is not entirely improbable.

We did not, however, recover any early generation hybrids (i.e., <F3). The absence of such early-stage hybrids suggests that hybridization is not frequent. We note that all six putative hybrids were either backcrosses from or have introgression from the most populous species, the Mourning Dove. Although it is difficult to extrapolate from such a small sample, this may suggest that introgression in this system fits Hubbs’ Principle (a.k.a., ‘Desperation hypothesis’ [90]) and may only occur in extreme circumstances. Additionally, the lack of mtDNA introgression in these birds is consistent with Haldane’s Rule, which predicts that female hybrids have lower fitness than males in ZW systems [91]. Evidence of Haldane’s Rule has been found previously in Columbiformes (i.e., Rock Pigeon [32,92,93]), and may implicate strong female mate choice, controlled copulation, or lower viability of female hybrids in limiting hybridization in these birds. Regardless of the mechanisms, however, it is clear that despite the present extensive geographic overlap of these four large doves in North America, hybridization is rare and unlikely to lead to significant gene flow between these four species.

### 4.3. Demographic Histories Differentially Impact the Genomes of the Four Dove Species

We found that all four dove species maintained similar population sizes until approximately nine million years ago (Figure 6). Given that these species likely diverged within their respective sub-clades during the mid to late Miocene [39,78,79,80,94,95], our demographic analyses suggest that they likely retained the ancestral state for several million years before expanding. Subsequently, we identified species-specific patterns. Rock Pigeon and Eurasian Collared-Dove appear to show cyclical patterns of expansion and contraction during the Pliocene, followed in Rock Pigeon by a recent increase. Mourning and White-winged Doves instead show a pattern of gradual recent increase. These distinct trajectories have culminated in different levels of current genetic diversity in the four species, with many estimates indicating Rock Pigeon has high diversity relative to the other species (Table 1). In general, these patterns of *N_e_* change are similar to those in other avian species that have been linked to large global climate events since the Miocene (e.g., glaciations [96]), including other New World Columbiformes like the Passenger Pigeon (*Ectopistes migratorius* [97,98]) and Band-tailed Pigeon (*Patagioenas fasciata* [98]). The similarity in the patterns in the two species native to the Americas may reflect similar responses to recent climate amelioration in North America.

It is important to highlight that estimates of genetic diversity and effective population size differ within our study, and also between our study and prior work. As with divergence times, some of these differences likely differ due to marker type. In our results, for example, Mourning Dove had the highest autosomal nuclear diversity, but Rock Pigeon had the highest diversity in mtDNA and sex-linked loci. Other differences are likely methodological in nature. Specifically, values estimated by ∂a∂i in our single species analyses differed from those in our pairwise species analyses. We argue in this case that, because the single species models had significantly improved model fit over pairwise analyses, these likely reflect the most accurate assessment of effective population size through time. Finally, the sampling of individuals likely also played a role in some differences we observed. For example, nucleotide diversity of Eurasian-Collared doves in our study was an order of magnitude less than that identified in European, Asian and Caribbean populations (avg. π = 0.026 [99]) but greater than that in Japan (avg. π = 0.00077 [100]). These differences may derive from the different geographic regions sampled across these studies. Similarly, we recovered an increasing *N_e_* over the last 1.1 million years for Rock Pigeons, while previous work using full genomes finds a sharp decline to low or near-zero levels (~500,000 [96,100]). However, these genome-based studies included just a few or only one individual, often obtained directly from domestic settings [96,100]. The precipitous decline in effective population size they identified in Rock Pigeon could be attributable to uncertainty resulting from the small sample size coupled with the effects of inbreeding in captivity. We also caution, however, against over-interpretation of our effective population size results for Rock Pigeons and Eurasian Collared-Doves based on individuals sampled in North America. These populations are not only introduced into North America, but from domesticated stock [12,15]. Founder events and domestication likely have resulted in reduced genetic diversity, which is known to bias demographic analyses, particularly at more recent time estimates [96,101]. We suggest that the sampling of individuals be carefully considered in studies of historical demography and also recommend approaches, like the novel step-wise methods presented here, that integrate information across multiple individuals.

## 5. Conclusions

Overall, our results indicate that substantial genome-wide divergence is present in the four large doves of southwestern North America, and that recent widespread sympatry in the region has not led to rampant anthropogenic hybridization. Although the four species exhibit distinct demographic trajectories in the past, further monitoring will clarify whether current and future populations continue on these same trends. Future work will also benefit from increasing geographic and genomic sampling of these doves to determine if hybridization rates vary geographically or demographic inferences differ depending on the geographic origins of sampled individuals.

## Figures and Tables

**Table 1:** Nucleotide diversity and effective population size (N_E_) estimates.

	mtDNA COI Nucleotide Diversity (π)	ddRAD-Seq Z-Sex Nucleotide Diversity (π)	ddRAD-Seq Autosomal Nucleotide Diversity (π)	∂a∂i ddRAD-Seq Autosomal Based N_E_ (Millions) Paired Species	∂a∂i ddRAD-Seq Autosomal Based N_E_ (Millions) Single Species	Census (Millions)
Eurasian Collared-Dove	0.0024	0.0064	0.0088	0.60	2.95	85 ^1^
Rock Pigeon	0.00084	0.0073	0.0081	0.95	9.03	140 ^1,2^
Mourning Dove	0.00072	0.015	0.023	1.22	5.08	249 ^1,3^
White-winged Dove	0.00057	0.0062	0.0089	1.04	5.58	14 ^1^

^1^: Partners in Flight. In *Avian Conservation Assessment Database*. 2017. Available online: http://pif.birdconservancy.org/acad/database.aspx (accessed on 28 April 2020). ^2^: Allison, A.B.; Mead, D.G.; Gibbs, S.E.; Hoffman, D.M.; Stallknecht, D.E. West Nile virus viremia in wild rock Rock Pigeon. *Emerg. Infect. Dis.* **2004**, *10*, 2252–2255. ^3^: Seamans, M.E. *Mourning Dove Population Status*; U.S. Department of the Interior, Fish and Wildlife Service, Division of Migratory Bird Management: Laurel, Maryland, 2019.

## Data Availability

Mitochondrial DNA sequences: GenBank accession numbers OK086092–OK086272. Illumina ddRAD-Seq Reads: NCBI’s Sequence Read Archive data BioProject PRJNA761761: BioSample Accession Numbers SAMN21362192–SAMN21362373.

## Supplementary Materials

The following are available online at https://www.mdpi.com/article/10.3390/ani11092677/s1, Table S1: Sample information, Table S2: ∂a∂i model likelihood comparisons for pair-wise species comparisons, Supporting Information Document S1: Detailed ddRAD-Seq library preparation methods and detailed ∂a∂i methods to estimate effective population size, divergence time, and migration rates, Figure S1: Total number of base-pairs recovered across nuclear chromosomes, Figure S2: Genetic based sex identification across samples, Figure S3: Posterior distributions of pair-wise species migration rates as estimated in IM using the mtDNA COI gene, Figure S4: Posterior distributions of pair-wise species ancestral and per species effective population sizes as estimated in IM using the mtDNA COI gene, Figure S5: Posterior distributions from pair-wise species ancestral and per species effective population sizes as estimated in IM using the mtDNA COI gene, Figure S6: Optimum ∂a∂i models and their associated SFS and likelihoods across single species demographic and pair-wise species analyses.
