# Supplementary material for: Genomic Evidence for Rare Hybridization and Large Demographic Changes in the Evolutionary Histories of Four North American Dove Species
Source: Animals (Basel). 2021 Sep 13;11(9):2677. doi: 10.3390/ani11092677 (PMC8468798; doi:10.3390/ani11092677)
Supplement: Supplementary file 1 [file animals-11-02677-s001.zip › RAD.DOV.Supp.Info.Doc.S1.pdf]

Detailed Mitochondrial DNA PCR and Sanger Sequencing

A 500-base pair (bp) fragment of the mtDNA cytochrome oxidase subunit 1 (COI) gene was amplified and sequenced across dove samples in Eurasian Collared-Dove, White-winged Dove, and Mourning Dove using newly designed primers DOVR (5'-GGTTKCGGTCYGTRAGTAG-3') and DOVF (5'-RGGAGAYGACYMAATCTMYA-3'). Additional primers were also designed for Rock Pigeon PigR (5'-AGGTTTCGGTCTGTGAGCAG-3') and PigF (5'-CCTCCTCATCCGAGCAGAAC-3'). PCR solutions included 1.5 µl of template DNA (≥10 ng/µl), 2x GoTaq Green Master Mix (Promega), and 1.0 nM of each primer, in a total volume of 15µl. Next, we optimized a touchdown PCR protocol that enabled amplification for each species using the DOVR/F primers. Thermocycler conditions included an initial denaturation at 94°C for 7 min, followed by a single 94°C cycle for 20 sec, before annealing for another 20 sec starting at 62°C decreasing by one degree each cycle to 52°C, and a final 1minute extension at 72°C. This touch-down PCR protocol was followed by 30 cycles of 20 sec at 94°C, 20 sec at 52°C, and 1 min at 72°C, with a final extension at 72°C for 10 minutes. Note the annealing temperature for the PigR/F primer pair followed the same PCR conditions but the annealing step was simply kept at 62°C for 45 cycles. Amplification was verified using gel electrophoresis with a 1% agarose gel. PCR products were cleaned with 4.0 µl of a 10-fold diluted solution of ExoSAP-IT® (USB Corporation, Cleveland, OH) and 6 µl of PCR product. PCR cleanup followed an extended protocol of 37°C for 30 min and 80°C for 15 min. Final products were then sequenced on a 3130XL Genetic Analyzer at the University of Texas El Paso, Border Biomedical Research Center's Genomic Analysis Core Facility. Raw Sanger sequences were aligned and edited using SEQUENCHER v4.8 (Gene Codes, Inc). All sequences have been submitted to GenBank (Accession Numbers *TBD*).

Detailed ddRAD-Seq library preparation methods

ddRAD-seq libraries were prepared following protocols of [1]; also see [2,3], but with modification when size selecting. First, ~0.2 ng of genomic DNA was digested with 10 U each of Sbfl and EcoRI restriction enzymes, followed by ligating adapters containing sequences compatible for Illumina TruSeq reagents and barcodes for de-multiplexing. Adapter-ligated DNA fragments were then subject to double sided size selection based on a 0.8x solution of AMPure XP beads (Beckman Coulter, Inc.). In short, right-sided selection for large fragments was first accomplished by adding a 0.55x concentration of the total starting ligated DNA solution. This solution was allowed to sit for 10 minutes, before being transferred to a magnetic plate to rest for 5 minutes. The supernatant, which contains target-sized and small-sized fragments, was then transferred to new tubes and the beads were discarded. Next, a left-sided size selection against fragments of < 100bp was done by adding another bead solution at 0.25x concentration of the total starting ligated DNA and let stand at room temperature for 10 minutes. Subsequently, tubes were moved back onto a magnetic plate and rested for 5 minutes at room temperature. The supernatant containing <100bp fragments was then discarded and the beads were washed twice with 80% ethanol. The beads were then air-dried at room temperature for 15 minutes but monitored to take care not to over dry. DNA was re-suspended with 25 μL ddH2O and eluted for a minimum of 30 minutes before proceeding. Size selected fragments were then PCR amplified with Phusion High-Fidelity DNA Polymerase (Thermo Scientific), and 10x concentration of forward and reverse primers for which compatible sequences are present on our barcodes and indices (also see [1]), and under the following PCR conditions: an initial 30 sec cycle at 98°C, followed by 22 cycles of 10 sec at 98°C, 30 sec at 60°C, and 40 sec at 72°C, with a final extension at 72°C for 5 min. Amplified products were then cleaned using a 1.8x solution

of AMPure XP magnetic beads (Beckman Coulter, Inc) and two 80% ethanol washes before a final elution in 40µl of ddH_2O. Library concentrations across samples were quantified using a Qubit dsDNA BR Assay Kit following manufacturers protocols. Samples were then pooled in equimolar amounts, and the multiplexed library was sequenced on an Illumina HiSeq X using single-end 150 bp chemistry with NovoGene (Novogenetics Co., Ltd.).

Detailed Nuclear Population Structure Analyses

Population structure & Diversity statistics

For nuclear DNA, population structure was based on bi-allelic SNPs and with all analyses carried out without *a priori* assignments. First, we used PLINK v1.07 [4] to filter bi-allelic datasets for singletons (i.e., minimum allele frequency (--maf 0.0055)), any SNP missing ≥20% of data across samples (--geno 0.2), as well as any SNPs found to be in linkage disequilibrium (LD) (--indep-pairwise 2 1 0.5). If an LD correlation factor (r^2) > 0.5 was obtained, one of the two SNPs was randomly excluded. We performed Principle component analysis (PCA) as implemented in the package adegenet in R (i.e. "dudi.pca"; [5]; also see [6]). Next, we calculated maximum-likelihood-based individual assignment probabilities using the program ADMIXTURE v1.3.0 [7,8]. SNP datasets were formatted for ADMIXTURE analysis, then processed through PLINK v1.07 [4] following steps outlined in [9]. For each ADMIXTURE analysis, a 10-fold cross-validation was performed, with a quasi-Newton algorithm employed to accelerate convergence [10]. For each number of populations (*K* = 1-10) tested, we used a block-relaxation algorithm for the point estimation, with analyses terminated once the change (i.e. delta) in the log likelihood of the point estimation increased by <0.0001. The optimum K was based on the lowest average of CV-errors across 100 analyses per evaluated *K* value. We used

the R package PopHelper [11] to convert ADMIXTURE outputs into CLUMPP input files at each K. We determined the robustness of individual assignments to populations at each K employing the Greedy algorithm and 1000 random permutations in the program CLUMPP V.1.1 [12]. Final outputs were based on ADMIXTURE proportions (Q estimates; the log likelihood of group assignment) per individual. Finally, we tested for relationships and admixture among samples with the program fineRADstructure [13] that includes RADpainter v 0.1 and finestructure [14]. Briefly, fineRADstructure derives a co-ancestry matrix based on the distribution of identical or nearest neighbor alleles among samples. Co-ancestry of each individual at each locus is equally divided among all other individuals with identical haplotypes, or in the case of unique alleles all other individuals with the "nearest neighbor" haplotype. Rare haplotypes defined by rare SNPs, which are on average of more recent origin, make the greatest contribution to the resulting pairwise co-ancestry coefficients, providing a measure that emphasizes recent co-ancestry. This analysis is completed without a priori information on population or species identity. The analysis was run with a burn-in of 100,000 iterations, followed by 100,000 Markov chain Monte Carlo (MCMC) steps, and with default parameters. Results were visualized using the R scripts fineradstructureplot.r and finstructurelibrary.r (available at http://cichlid.gurdon.cam.ac.uk/fineRADstructure.html).

Detailed Phylogenetic Analyses

First, a mtDNA species tree was reconstructed in *Beast v2.6.0 [15] using a multispecies Calibrated Yule tree method (Species Tree: Yule Process). Beast employs a MCMC to estimate posterior distribution of the final species tree given a set of gene trees. We rooted the species tree using a Mallard COI mtDNA sequence (https://www.ncbi.nlm.nih.gov/nuccore/MK262361.1#).

Supporting Information Document S1

An optimum base-pair substitution model was determined based on Bayesian Information Criterion (BIC) scores estimated in MEGA v10 [16]. Furthermore, we tested strict and variable molecular clocks by comparing Bayes Factors estimated from respective reconstructed species trees. A model with a Bayes Factor of ≥ 3 was considered to be the optimum model [17]. The split between Mallards and Rock Pigeon was inferred to be 93.2-104.6 mya [18], and thus, a lognormal distribution prior was used on this node with a minimum age 90.6 mya (2.5% quantile) and maximum age 94.4 mya (97.5% quantile; mean = 0.5, standard deviation = 0.5, offset =90.0). Lognormal distributions were applied to the remaining prior subsets (i.e. birth rate, clock rate, freqParameter kappa, and popMean). Beast analyses comprised one concurrent run of 500 million MCMC generations with sampling every 5,000 generations. Log files were inspected to ensure that effective sample size (ESS) values across estimated parameters were >50. The first 10% of trees were discarded as burn-in using TreeAnnotator v2.6.2, and a final tree was summarized in Tracer v.1.7.1. The final species tree was visualized in FigTree v1.4.4 (http://tree.bio.ed.ac.uk/software/figtree).

Next, a species tree based on ddRAD-seq loci was reconstructed in the program TreeMix version 1.12 [19], using a Mallard as the tree root. We used only bi-allelic SNPs that were formatted and filtered for singletons (i.e., minimum allele frequency (--maf 0.0055)), and missing data (>15% missing data removed; --geno 0.15) in the program PLINK v1.07 [4]. We then filtered for linkage disequilibrium (LD; --indep-pairwise 2 1 0.5), randomly retaining one of the SNPs found to be in LD. In addition to establishing phylogenetic relationships, TreeMix was used to test for gene flow in a phylogenetic context. Specifically, TreeMix simultaneously estimated a maximum likelihood (ML) species tree and the direction and weight (*w*) of gene flow among taxa based on allele frequencies. Analyses were run across each bi-allelic SNP (-k 1),

with global rearrangement occurring during tree building (-global). Node support was based on 1,000 bootstraps using the python script *treemix_tree_with_bootstraps.py* (https://github.com/mgharvey/misc_python/blob/master/bin/TreeMix/treemix_tree_with_bootstraps.py). A final species tree and nodal support was summarized across bootstraps using TreeAnnotator v2.5.2 and viewed in FigTree v1.4.0 (http://tree.bio.ed.ac.uk/software/figtree). Next, analyses were run for up to ten migration events (-m 0 - 10), and with the optimum number of migration edges determined by the proportion of the variance explained by each migration model, and estimated with the 'get_f()' R function provided with the TreeMix Package. In order to limit overconfidence in the tree model, migration edges were added until >98% of the variance in the tree model was explained. Finally, likelihood ratios and associated critical-X^2 p-values were calculated to assess significance between tree models with and without gene flow. Standard errors (-se) calculated in TreeMix were used to assess statistical significance of each migration edge.

Detailed methods in estimates of effective population size, divergence time, and migration rates

For mtDNA, isolation-with-migration models as implemented in IM [20,21] were used to estimate effective population size, divergence time, and migration rates for pairwise comparisons of all four species. In short, IM simultaneously calculates posterior probability densities of population sizes, divergence time, and migration rates from non- recombinant sequence fragments using Bayesian MCMC algorithms [22]. IM analysis were run for a minimum of 10,000,000 generations following a burn-in of 1,000,000 generations or until the effective samples sizes (ESS) were $\geq$ 50 across all parameters [20,21]. Migration rates (*m*), time since

divergence (*t*), and the effective population size of the ancestor (Ne_{ANC}), Mourning Dove (Ne_{MODO}), White-winged Dove (Ne_{WWDO}), Eurasian Collared-Dove (Ne_{EUCD}) and Rock Dove (Ne_{ROPI}) were estimated.

For nuclear DNA, we used the program ∂a∂i (Diffusion Approximations for Demographic Inference [23]) to simultaneously estimate rates of gene flow, time since divergence, and effective population sizes within and between each species. In short, ∂a∂i uses site frequency spectrum (SFS) data and implements a diffusion approximation-based approach to fit specified evolutionary models (e.g., isolation-with-migration) against empirical data [23,24]. Given that ∂a∂i is most accurate and efficient when working with only two populations [25], we calculated the best fit model for each species-by-species comparison. Briefly, we can determine the best fit model by using the multinomial estimate of log-likelihood for optimized parameters that best fit the model SFS to the empirical SFS. After concatenating all loci in a Nexus file format, we used custom python scripts (https://github.com/jibrown17/Dove_dadi.pairwise.comparisons) to derive an empirical SFS using ∂a∂i. Each SFS was based on only minor alleles and was folded because we lacked outgroup information [23]. Additionally, variants observed in none or all samples were ignored (‘masked’) by ∂a∂i. Finally, to account for missing data and the lack of shared variants between species, each SFS was projected down to create the most complete SFS, while maintaining the most alleles possible [24]. Given that relatedness between each species varies, projections for each species-by-species comparison differ in order to account for the low number of shared variants. We used the following datasets: (1) Eurasian Collared-Dove (N = 20 alleles) x Mourning Dove (N = 15 alleles), (2) Eurasian Collared-Dove (N = 23 alleles) x Rock Dove (N = 23 alleles), (3) Eurasian Collared-Dove (N = 18 alleles) x White-winged Dove (N = 18), (4)

Rock Dove (N = 20 alleles) x Mourning Dove (N = 15), (5) Rock Dove (N = 30 alleles) x White-winged Dove (N = 10), (6) White-winged Dove (N = 17 alleles) x Mourning Dove (N = 17 alleles).

For each dataset, we tested the empirical data against three evolutionary models that are included in the program ∂a∂i : (1) Neutral-No-divergence, (2) Isolation-With-Migration, and (3) Split-With-Migration (https://github.com/jibrown17/Dove_dadi.pairwise.comparisons). We also tested the data against custom Split-No-Migration and Isolation-No-Migration models where we forced zero migration in the standard models. We determined which model best fit the empirical SFS based on the highest log-likelihood of the optimal parameters for each model. We then performed 20 independent parameter optimizations of the best fit model for each species comparison. Different demographic parameters were estimated for each model, including a scaling factor ($\theta = 4N_{ANC}$ x μ; N_{ANC} = Ancestral effective population size), effective population sizes ($N_i = v_i$ x N_{ANC}), migration rates ($m_{i\leftarrow j} = M_{i\leftarrow j}/(2N_{ANC})$; $m_{i\leftarrow j}$ = proportion of migrants/generation in population i from population j), and time since divergence ($t = T$ x $2N_{ANC}$; t = time since divergence in generations; [24]).

To convert parameter estimates from IM and ∂a∂i into biologically informative values, we estimated generation time (G) and mutation rates per locus (μ). First, generation time (G) is calculated as $G = \alpha + (s/(1-s))$, where α is the age of maturity and is the expected adult survival rate [26]. For all species comparisons we used an age of maturity (α) of 1 [27–30]. Additionally, for each species comparison we used the average of the adult survival (s) between the two species: Eurasian Collared-Dove x Mourning Dove (s = 0.565; [31,32]); Eurasian Collared-Dove x Rock Dove (s = 0.558; [31,33]); Eurasian Collared-Dove x White-winged Dove (s = 0.584; [31,34]); Rock Dove x Mourning Dove (s = 0.483; [32,33]); Rock Dove x White-winged Dove (s

= 0.502; [33,34]); White-winged Dove x Mourning Dove (s = 0.509; [32,34]). Finally, to obtain a scaled mutation rate for mitochondrial markers, we used an average mitochondrial mutation rate in birds of 1.035x 10^{-8} substitution/site/year [35]. For nuclear DNA, the mean avian nuclear mutation rate of 1.2 x 10^{-9} substitutions/site/year [36] is unlikely to be representative of our four species (i.e., Columbiaformes tend to show slower mutation rates; [37]) or isolated ddRAD-seq markers. In particular, given the high divergence recovered with nuclear and mtDNA loci across the four dove species (Table 1; Fig. 2C). we expected overlapping ddRAD-seq loci that are unbiased from complete or partial allelic dropouts to be more conserved, and thus, having a slower mutation rate. Thus, instead, we determined a mutation rate by setting the autosomal divergence time as calculated by ∂a∂i to the average of inferred time splits within the *BEAST analysis of mtDNA for Eurasian (i.e., Eurasian Collard-Doves and Rock Pigeons) and North American (White-winged and Mourning Doves) clades (see results; also see [38]). In short, we used the derived average mtDNA time to set the ∂a∂i time parameter, and thus calibrating the N_{ANC} parameter. Doing so, we then derive a mutation rate (μ) using the formula $N_{ANC} = \theta/4\mu$. These mutation rates were then scaled to the generation times for each species comparison before being multiplied by the total number of base pairs for mtDNA (419 bp) and nuclear DNA (79,862 bp) to get substitutions/site/generation (s/s/g); and which were then used to convert all respective parameter values. Ancestral effective population size was derived as $N_{ANC} = \theta / \mu$ for mtDNA and $N_{ANC} = \theta/4\mu$ for nuclear DNA, where θ is scaled to the mutation rate per generation [39]. Effective population size was derived as $N_E = \theta_{IM}/4\mu$ for mtDNA and $N_E = v_E$ x N_{Anc} for nuclear DNA. Finally, years since divergence (T) was then derived for mtDNA as $T = t/\mu$, where t is the time since divergence scaled to geometric mean of the mutation rate (μ) [20], and

calculated in ∂a∂i using the following formula $t_n = T_n$ x 2 x N_{ANC} x G (t_n = total years before present at the nth time interval & G = generation time; Sæther et al. 2005).

Detailed methods on Historical population demography through time

Long-term demographic histories of each species were determined using a novel ∂a∂i model that estimates effective population size through time using partial genome sequence information. Using the same set of bi-allelic ddRAD-seq SNPs as previously described, we created a one-dimensional (i.e., single species) site-frequency spectrum (SFS) for each species where Nexus formatted ddRAD-seq autosomal loci are transformed into species-specific SFS using custom python scripts (https://github.com/jibrown17/Dove_dadi.demographics). The SFS was then folded and masked at sites with variants present in only one or all samples [23,24]. Datasets were projected down to account for missing data between samples (N_{ECDO} = 37 alleles, N_{MODO} = 61 alleles, N_{ROPI} = 28 alleles, N_{WWDO} = 59 alleles). Each dataset is then run through our custom model (https://github.com/jibrown17/Dove_dadi.demographics) where effective population size is estimated through a series of time intervals. In short, our stepwise time interval function uses 100 iterations of the single population integration function ('*Integration.one_pop*' in ∂a∂i) to model a continuous transformation of effective population size through time.

This model starts by estimating the ancestral effective population size of v_{Anc}, which exists for some time-period, T_{Anc}, before estimating the effective population size, v_n, for some time interval, T_n, at each subsequent integration step. Effective population size is then estimated for time intervals in the past, starting with T_0, until present day, T_{99}, and the ancestral population will have occurred at time, $T_{99} + T_{98} + T_{97} \ldots + T_1 + T_0 + T_{Anc}$. This stepwise function is then used to model an SFS that is subsequently fit to the empirical data for each species through parameter

optimization. Optimum parameters are estimated by performing 50 independent parameter optimization runs, with each optimization run having a random starting point. Specifically, for parameter optimization, we first extrapolated the stepwise function of continuous population change across a grid in frequency spectrum space that is used by ∂a∂i to infer a final model SFS. Next, we randomize initial input values for optimization using the ∂a∂i function '*perturb_params*', before finally calculating parameter optimums (i.e., v_{Anc}–v_{99} & T_{Anc}–T_{99}) with the function '*optimize_log*'. While starting points for optimization are randomized, time interval parameters are constrained based on known estimates of time since divergence from the pairwise species comparison results. Final optimum parameters are scaled to the empirical data using θ ($\theta = 4N_{ANC} \times \mu$; N_{ANC} = Ancestral effective population size). Finally, we estimate the goodness of fit for each species' model SFS by calculating the log-likelihood of the model given the empirical data, as well as visualize model fit by graphing model residuals.

Next, 95% confidence intervals (CI) were estimated using the parameter uncertainty metrics included in ∂a∂i. Briefly, ∂a∂i calculates uncertainty values using a Fisher Information Matrix (FIM), which provides a measure of how much information can be derived from the data with respect to an unknown parameter. This FIM can then be used by ∂a∂i to calculate variance and uncertainty of that parameter. The FIM requires a step size (ε) to be chosen for the calculation of the numerical derivatives [24,40,41]. We note that ∂a∂i is unable to calculate an uncertainty value for a parameter if the numerical derivative of the parameter is negative; therefore, we maximized the number of parameters for which ∂a∂i is able to return a true estimate of uncertainty by calculating uncertainty across a range of step sizes ($\varepsilon = 10^{-2} – 10^{-7}$; [24,40,41]).

Finally, ∂a∂i parameters were converted into biologically informative numbers as previously described and based on generation time (*G*) that is calculated using an age of sexual maturity (α) of one for each species, as well as survival estimates specific to Rock Pigeon (s = 0.48; [33]), Eurasian Collared-Dove (s = 0.64; [31]), Mourning Dove (s = 0.49; [32]), and White-winged Dove (s = 0.53; [34]). Similarly, we derived substitutions per generations by scaling our derived mutation rate of 1.95×10^{-10} mutations/site/year to each individual species' generation time and total ddRAD-seq sequence length. In short, the ∂a∂i parameter of time before present is converted into years based on the formula $t_n = T_n \times 2 \times N_{Anc} \times G$ (t_n = total years before present at the nth time interval & G = generation time), and the effective population size calculated for each time interval as $N_n = v_n \times N_{Anc}$ (N_n = effective population size at the nth time interval).

REFERENCES

1. Dacosta JM, Sorenson MD. 2014 Amplification Biases and Consistent Recovery of Loci in a Double-Digest RAD-seq Protocol. *PLoS One* **9**, e106713. (doi:10.1371/journal.pone.0106713)
2. Lavretsky P, McInerney NR, Mohl JE, Brown JI, James HF, McCracken KG, Fleischer RC. 2020 Assessing changes in genomic divergence following a century of human-mediated secondary contact among wild and captive-bred ducks. *Mol. Ecol.* **29**, 578–595. (doi:10.1111/mec.15343)
3. Lavretsky P, Janzen T, McCracken KG. 2019 Identifying hybrids & the genomics of hybridization : Mallards & American black ducks of Eastern North America. *Ecol. Evol.* , 1–21. (doi:10.1002/ece3.4981)
4. Purcell S *et al.* 2007 PLINK : A Tool Set for Whole-Genome Association and Population-

Based Linkage Analyses. *Am. J. Hum. Genet.* **81**, 559–575. (doi:10.1086/519795)

5. Dray S, Dufour A-B. 2007 The ad4 Package: Implementing the Duality Diagram for Ecologist. *Jounal Stat. Softw.* **22**, 1–20.
6. Jombart T, Lyon D, Biome L De. 2008 adegenet : a R package for the multivariate analysis of genetic markers. *Bioinformatics* **24**, 1403–1405. (doi:10.1093/bioinformatics/btn129)
7. Alexander DH, Lange K. 2011 Enhancements to the ADMIXTURE algorithm for individual ancestry estimation. *BMC Bioinformatics* **12**, 246. (doi:10.1186/1471-2105-12-246)
8. Alexander DH, Novembre J, Lange K. 2009 Fast model-based estimation of ancestry in unrelated individuals. *Gennome Res.* **19**, 1655–1664. (doi:10.1101/gr.094052.109.vidual)
9. Alexander DH, Lange K. 2015 Admixture 1.3 Software Manual.
10. Zhou H, Alexander D, Lange K. 2011 A quasi-Newton acceleration for high-dimensional optimization algorithms. *Stat Comput* , 261–273. (doi:10.1007/s11222-009-9166-3)
11. Francis RM. 2016 POPHELPER: an R package and web app to analyse and visualize population structure. *Mol. Ecol. Resour.* **17**, 27–32. (doi:10.1111/1755-0998.12509)
12. Jakobsson M, Rosenberg NA. 2007 CLUMPP : a cluster matching and permutation program for dealing with label switching and multimodality in analysis of population structure. *Bioinformatics* **23**, 1801–1806. (doi:10.1093/bioinformatics/btm233)
13. Malinsky M, Trucchi E, Lawson DJ, Falush D. 2018 RADpainter and fineRADstructure : Population Inference from RADseq Data. *Mol. Biol. Evol.* **35**, 1284–1290. (doi:10.1093/molbev/msy023)
14. Lawson DJ, Hellenthal G, Myers S, Falush D. 2012 Inference of Population Structure

using Dense Haplotype Data. *PLoS Genet.* **8**, 11–17. (doi:10.1371/journal.pgen.1002453)

15. Drummond AJ, Suchard MA, Xie D, Rambaut A. 2012 Bayesian Phylogenetics with BEAUti and the BEAST 1 . 7. *Mol. Biol. Evol.* **29**, 1969–1973. (doi:10.1093/molbev/mss075)

16. Tamura K, Ursula F, Billing-ross P, Murillo O, Filipski A, Kumar S. 2012 Estimating divergence times in large molecular phylogenies. *PNAS* **109**, 19333–19338. (doi:10.1073/pnas.1213199109)

17. Kass RE, Raftery AE. 1995 Bayes Fcators. *J. Am. Stat. Assoc.* **90**, 773–795.

18. Kumar S, Stecher G, Suleski M, Hedges SB. 2017 TimeTree : A Resource for Timelines , Timetrees , and Divergence Times. *Mol. Biol. Evol.* **34**, 1812–1819. (doi:10.1093/molbev/msx116)

19. Pickrell JK, Pritchard JK. 2012 Inference of Population Splits and Mixtures from Genome-Wide Allele Frequency Data. *PLoS Genet.* **8**, e1002967. (doi:10.1371/journal.pgen.1002967)

20. Hey J, Nielsen R. 2004 Multilocus methods for estimating population sizes, migration rates and divergence time, with applications to the divergence of Drosophila pseudoobscura and D. persimilis. *Genetics* **167**, 747–760. (doi:10.1534/genetics.103.024182)

21. Hey J, Nielsen R. 2007 Integration within the Felsenstein equation for improved Markov chain Monte Carlo methods in population genetics. *Proc. Natl. Acad. Sci. U. S. A.* **104**, 2785–2790. (doi:10.1073/pnas.0611164104)

22. Nielsen R, Wakeley J. 2001 Distinguishing Migration from Isolation: A Markov Chain Monte Carlo Approach. *Genetics* **158**, 885–896.

23. Gutenkunst R, Hernandez RD, Williamson SH, Bustamante CD. 2010 Diffusion Approximations for Demographic Inference: ∂a∂i. *Nat. Preced.* **5**, 1–1. (doi:10.1038/npre.2010.4594.1)

24. Gutenkunst RN, Hernandez RD, Williamson SH, Bustamante CD. 2009 Inferring the Joint Demographic History of Multiple Populations from Multidimensional SNP Frequency Data. *PLoS Genet.* **5**, e1000695. (doi:10.1371/journal.pgen.1000695)

25. Noskova, E., Ulyantsev, V., O'Brien, J., S. and Dobrynin P. 2018 GADMA: Genetic Algorithm for Automatic Inferring Joint Demographic History of Multiple Populations from AlleleFrequency Spectrum. *bioRxiv Prepr.*

26. Sæther BE *et al.* 2005 Generation time and temporal scaling of bird population dynamics. *Nature* **436**, 99–102. (doi:10.1038/nature03666)

27. Kigir ES, Sivachelvan SN, Kwari HD, Sonfada MN, Yahaya A, Thilza IB, Wiam IM. 2010 Gross And Microscopic Changes In The Gonads Of Male And Female Domestic Pigeon (Columbia Livia). *New York Sci. J.* **3**, 108–111.

28. Nice MM. 1920 Some Experiences with Mourning Doves in Captivity. *Proc. Oklahoma Acad. Sci.* **1**, 57–65.

29. Prinzinger R, Misovic A. 2010 Age-correlation of blood values in the Rock Pigeon (Columba livia). *Comp. Biochem. Physiol. - A Mol. Integr. Physiol.* **156**, 351–356. (doi:10.1016/j.cbpa.2009.07.028)

30. White LM, Mirarchi RE, Lisano ME. 1987 Reproductive Capability of Wild Hatching-Year Mourning Doves in Alabama. *J. Wildl. Manage.* **51**, 204–211.

31. Aebischer NJ. 1995 Investigating the effects of hunting on the survival of British pigeons and doves by analysis of ringing recoveries. *J. Appl. Stat.* **22**, 923–934.

(doi:10.1080/02664769524711)

32. Otis DL. 2002 Survival Models for Harvest Management of Mourning Dove Populations. *J. Wildl. Manage.* **66**, 1052–1063.

33. Kautz JE, Malecki RA. 1990 *Effects of Harvest on Feral Rock Dove Survival, Nest Success and Population Size*. Los Alamos National Laboratory (USA).

34. Hall JD. 2018 Factors influencing annual survival and recovery rates, primary feather molt, and hatch chronology of White-winged doves in Texas. Tarleton State University.

35. Liu D, Hunt M, Tsai IJ. 2018 Inferring synteny between genome assemblies: A systematic evaluation. *BMC Bioinformatics* **19**, 1–13. (doi:10.1186/s12859-018-2026-4)

36. Nam K *et al.* 2010 Molecular evolution of genes in avian genomes. *Genome Biol.* **11**, 1–17. (doi:10.1186/gb-2010-11-6-r68)

37. Nabholz B, Glémin S, Galtier N. 2009 The erratic mitochondrial clock: variations of mutation rate, not population size, affect mtDNA diversity across birds and mammals. *BMC Evol. Biol.* **9**, 1–13. (doi:10.1186/1471-2148-9-54)

38. Oswald JA, Harvey MG, Depaul RCR, Donna UF, Steven LD, Robb WC. 2019 Evolutionary dynamics of hybridization and introgression following the recent colonization of Glossy Ibis (Aves : Plegadis falcinellus) into the New World. *Mol. Ecol.* **28**, 1675–1691. (doi:10.1111/mec.15008)

39. Peters JL, Zhuravlev YN, Fefelov I, Humphries EM, Omland KE. 2008 Multilocus phylogeography of a Holarctic duck: Colonization of North America from Eurasia by gadwall (Anas strepera). *Evolution (N. Y).* **62**, 1469–1483. (doi:10.1111/j.1558-5646.2008.00372.x)

40. Blischak PD, Barker MS, Gutenkunst RN. 2020 Inferring the Demographic History of

Inbred Species from Genome-Wide SNP Frequency Data. *Mol. Biol. Evol.* **37**, 2124–2136.

41. Coffman AJ, Hsieh PH, Gravel S, Gutenkunst RN. 2016 Computationally efficient composite likelihood statistics for demographic inference. *Mol. Biol. Evol* **33**, 591–593. (doi:10.1093/molbev)
