# Supplementary material for: Genomic Evidence for Rare Hybridization and Large Demographic Changes in the Evolutionary Histories of Four North American Dove Species
Source: Animals (Basel). 2021 Sep 13;11(9):2677. doi: 10.3390/ani11092677 (PMC8468798; doi:10.3390/ani11092677)
Supplement: Supplementary file 1 [file animals-11-02677-s001.zip › RAD.DOV.Supp.Mat.Table.S2.pdf]

Table S2. ∂a∂i model likelihood comparisons for pair-wise species comparisons between Rock Pigeons (ROPI), Eurasian Collard-dove (EUCD), White-winged Dove (WWDO), and Mourning Dove (MODO). Optimum models are bolded. Also, note that NaN means ∂a∂i was unable to calculate a likelihood value.

	∂a∂i Model Ln Likelihood				
	Neutral	IM	IM No-Mig	Split Mig	Split No-Mig
EUCD vs. ROPI	-3907.73	NaN	-717.40	**-471.47**	-1082.68
EUCD vs. MODO	-3121.82	-1102.48	-637.70	**-261.70**	-346.34
EUCD vs. WWDO	-5669.01	-492.09	-714.45	**-433.55**	-591.68
ROPI vs. MODO	-3083.43	-268.77	-368.49	**-243.47**	-353.94
ROPI vs. WWDO	-3900.69	NaN	-1179.32	-900.17	**-488.85**
MODO vs. WWDO	-1403.66	**-195.53**	-790.85	-210.92	-305.17
