# Supplementary figures and images for: Genomic Evidence for Rare Hybridization and Large Demographic Changes in the Evolutionary Histories of Four North American Dove Species
Source: Animals (Basel). 2021 Sep 13;11(9):2677. doi: 10.3390/ani11092677 (PMC8468798; doi:10.3390/ani11092677)

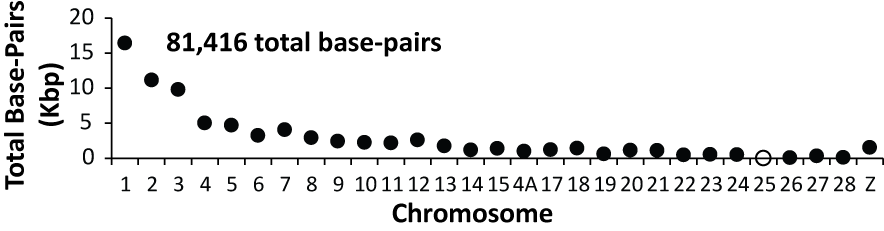

Supplement: Supplementary file 1 [file animals-11-02677-s001.zip › RAD.DOV.Supp.Info.FIG.S1.png]

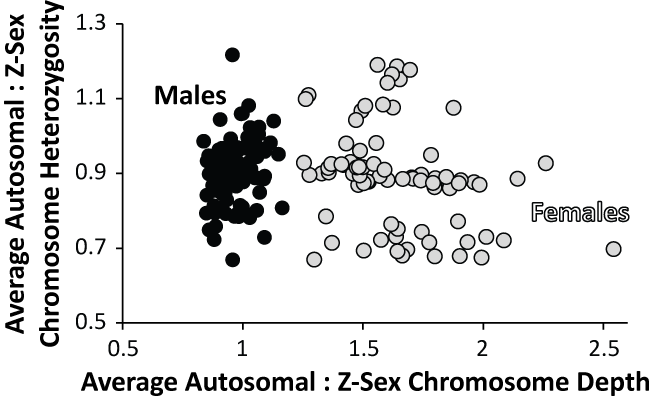

Supplement: Supplementary file 1 [file animals-11-02677-s001.zip › RAD.DOV.Supp.Info.FIG.S2.png]

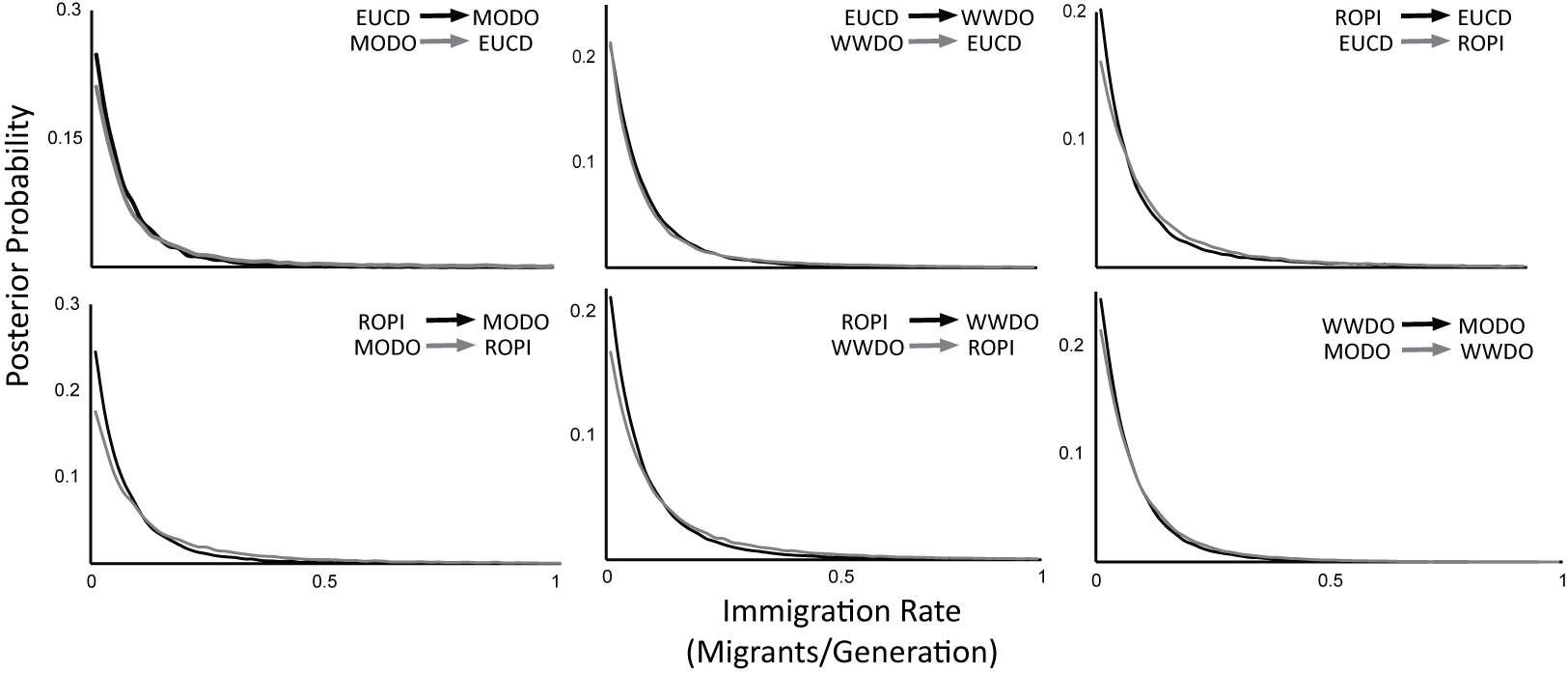

Supplement: Supplementary file 1 [file animals-11-02677-s001.zip › RAD.DOV.Supp.Info.FIG.S3.png]

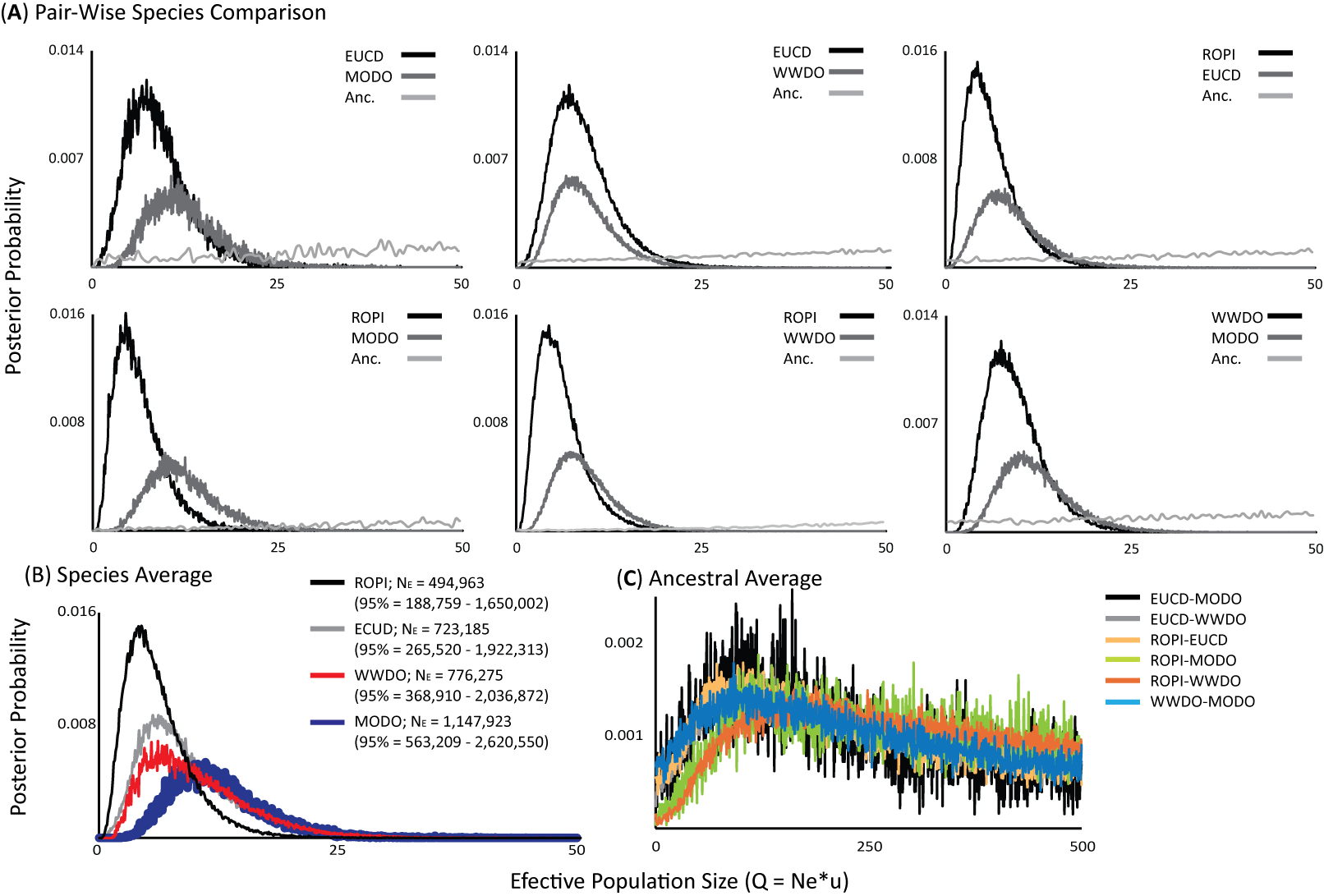

Supplement: Supplementary file 1 [file animals-11-02677-s001.zip › RAD.DOV.Supp.Info.FIG.S4.png]

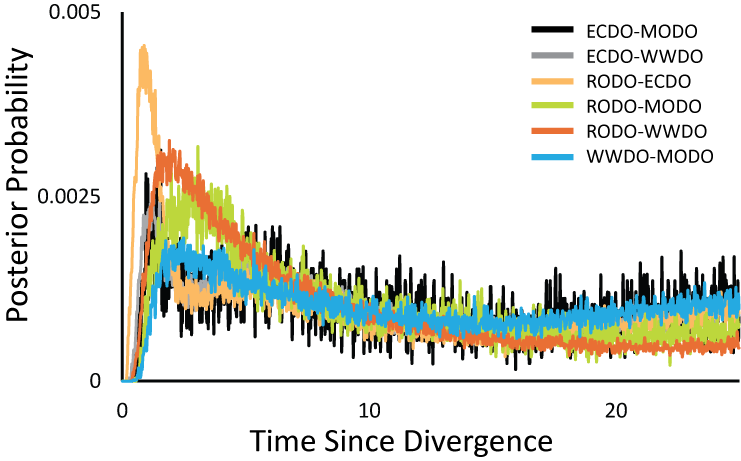

Supplement: Supplementary file 1 [file animals-11-02677-s001.zip › RAD.DOV.Supp.Info.FIG.S5.png]

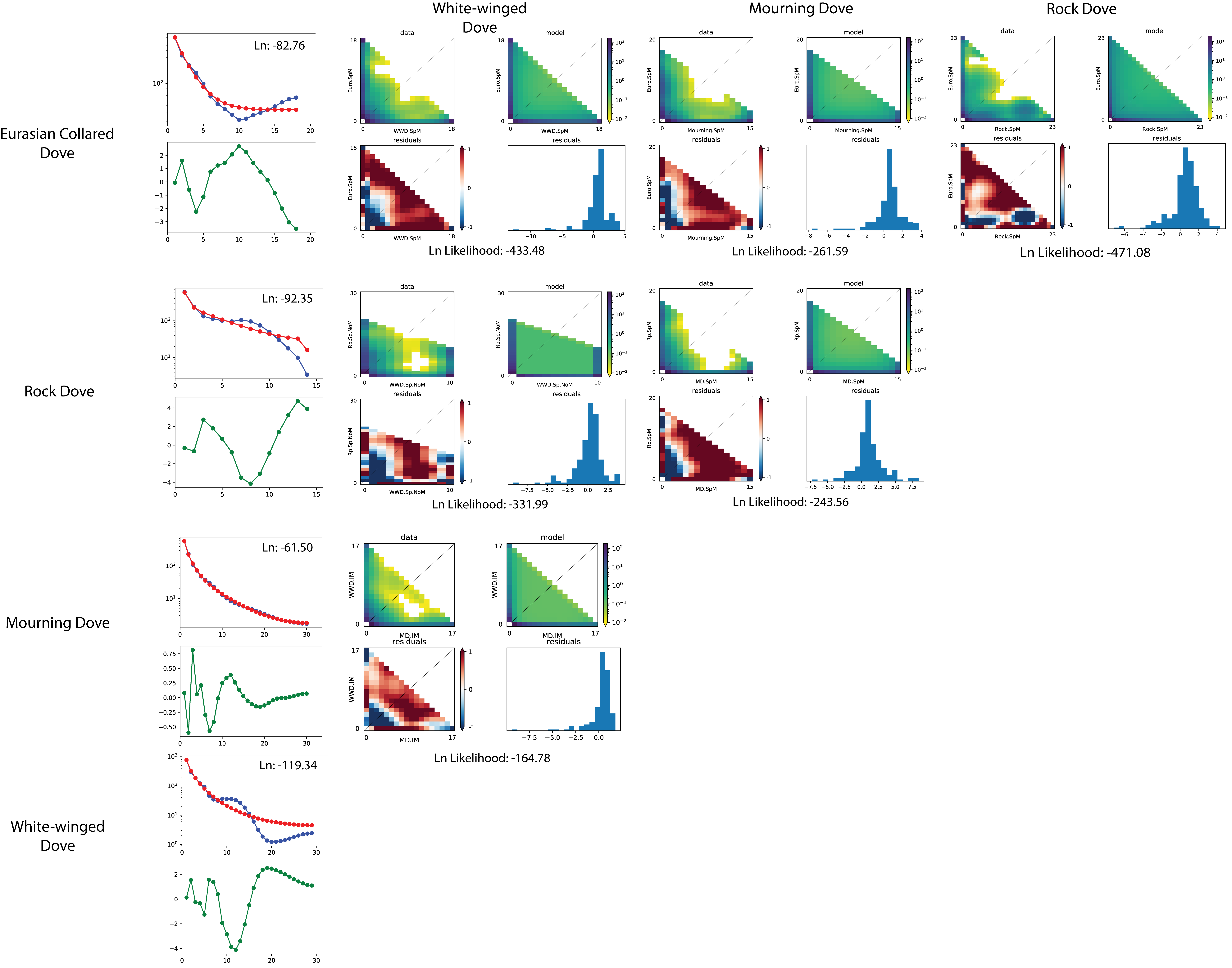

Supplement: Supplementary file 1 [file animals-11-02677-s001.zip › RAD.DOV.Supp.Info.FIG.S6.png]
